# Effects of ramosetron orally disintegrating tablets on the prophylaxis of post-discharge nausea and/or vomiting in female patients undergoing day surgery under general anesthesia: a randomized controlled trial

**DOI:** 10.1186/s13741-022-00251-6

**Published:** 2022-05-12

**Authors:** Hyun-Jung Shin, Yong-Hee Park, Minying Chang, Yun Jeong Chae, Hun-Taek Lee, Oh Haeng Lee, Sang-Kee Min, Sang-Hwan Do

**Affiliations:** 1 Department of Anesthesiology and Pain Medicine, Seoul National University Bundang Hospital, 82, Gumi-ro 173Beon-gil, Bundang-gu, Seongnam-si, Gyeonggi-do 13620 Republic of Korea; 2 Department of Anesthesiology and Pain Medicine, Chung-Ang University College of Medicine, Seoul, Republic of Korea; 3 Department of Anesthesiology and Pain Medicine, Ajou Medical Center, Soowon-si, Republic of Korea; 4 Seoul National University College of Medicine, Seoul, Republic of Korea

**Keywords:** Ramosetron, Post-discharge, Nausea, Vomiting, Outpatient surgery

## Abstract

**Background:**

This study was performed to evaluate the effectiveness of ramosetron orally disintegrating tablets (ODTs) in preventing post-discharge nausea and/or vomiting (PDNV) in female patients following outpatient surgery under general anesthesia.

**Methods:**

This multicenter randomized study included three South Korean tertiary hospitals. Before surgery, 138 patients were randomly allocated into two groups. In the ramosetron group, ramosetron ODT 0.1 mg was administered after discharge in the morning of postoperative days 1 and 2. Metoclopramide 10 mg was administered as a rescue antiemetic (capped at 30 mg per day). In the control group, patients were administered only metoclopramide 10 mg when nausea and/or vomiting occurred. The primary outcome was the incidence of nausea during 24 h after discharge.

**Results:**

We found significant differences in the incidence (13% vs. 33%, *P* = 0.008) and severity (*P* = 0.011) of nausea between the ramosetron and the control groups during 24 h after discharge. In addition, the rate of rescue antiemetic (metoclopramide) administration during 24 h after discharge was lower in the ramosetron group (6%) than in the control group (18%) (*P* = 0.033). Patient satisfaction score was higher in the ramosetron group than in the control group (*P* < 0.001).

**Conclusion:**

Ramosetron ODT reduces the incidence and severity of postoperative nausea after discharge during the first 24 h and may be a valuable option for the prevention of PDNV in female patients after day surgery under general anesthesia.

**Trial registration:**

ClinicalTrials.gov, NCT04297293. Registered on 05 March 2020

## Introduction

Approximately 20–30% of patients suffer from postoperative nausea and/or vomiting (PONV) after surgery (Gan et al. 2014). In some high-risk groups, the incidence of PONV is reported to be as high as 80% (Blacoe et al. 2008; Gan et al. 2007). One survey found that outpatients, especially those with a previous history of PONV, were even more willing to pay to avoid PONV (Gan et al. 2001).

More than 48 million procedures were performed on outpatients based in the USA in 2010 (Hall et al. 2017). With increasing numbers of surgical outpatients, post-discharge nausea and/or vomiting (PDNV) is an important clinical problem that needs to be addressed. One study reported that 37% of patients suffered from PDNV after ambulatory surgery under general anesthesia (Apfel et al. 2012). PDNV is particularly concerning in patients undergoing outpatient surgery as they cannot be administered rapid onset antiemetics intravenously at home, and they may not be able to tolerate oral medications.

Ramosetron is one of the selective 5-hydroxytryptamine (HT)_3_ receptor antagonists. Compared with ondansetron, which is one of the first generation 5-HT_3_ receptor antagonists, ramosetron has a significantly higher binding affinity and slower dissociation rate for 5-HT_3_ receptors. As a result, ramosetron is more potent and has a longer duration of action (Rabasseda 2002). The antiemetic effects of intravenous ramosetron or ramosetron orally disintegrating tablets (ODT) in the prevention and treatment of nausea and vomiting secondary to surgery or chemotherapy have been demonstrated in previous studies (Ryu et al. 2011; Sanmukhani et al. 2014). Importantly, ramosetron has an elimination half-life of 9 h, and the effects may last for up to 48 h (Hirata et al. 2007; Noda et al. 2002; Roh et al. 2014; Swaika et al. 2011). These properties of ramosetron make it potentially well-suited for the control of PDNV with a single oral daily dose. Nevertheless, clinical studies on the effect of ramosetron ODT in the prevention of PDNV are lacking in the literature.

Therefore, we undertook this study to evaluate the effectiveness of a single oral dose of ramosetron ODT in preventing PDNV in female patients undergoing outpatient surgery under general anesthesia. We hypothesized that ramosetron would have a prophylactic effect on PDNV after discharge from day surgery under general anesthesia.

## Methods

### Study setting

From May 2020 to March 2021, three South Korean tertiary academic hospitals participated in this prospective multi-center, randomized study (ClinicalTrials.gov ID NCT04297293), which was approved by the institutional review board of the following centers: Seoul National University Bundang Hospital (Gyeonggi, South Korea, IRB No. B-2002/594-003), Ajou University Medical Center (Gyeonggi, South Korea, IRB No. AJIRB-MED-INT-20-039), and Chung-Ang University Hospital (Seoul, South Korea, IRB No. 2060-003-420). Written, informed consents were obtained from all participants prior to their enrollment.

### Participants and trial design

Female patients (age range 18–49 years) with American Society of Anesthesiologists physical status 1 or 2 and scheduled for day surgery under general anesthesia were included in this randomized clinical trial. Patients who were pregnant or breastfeeding, taking other serotonin receptor antagonists, or had Lapp lactase deficiency, galactose intolerance, or glucose-galactose malabsorption prior to the study were excluded.

### Randomization

This was a parallel, block-randomized trial (block sizes of 6 and 8) with an allocation ratio of 1:1. The randomization table was created using a web-based randomization system (http://www.randomization.com). An anesthesiologist not involved in the study was in charge of randomization and prepared opaque, sealed envelopes containing a slip of paper with a computer-generated description of whether the patient would receive ramosetron ODT (ramosetron group) or not (control group).

### Blinding

The investigating anesthesiologist who performed the outcome assessment was not blinded to the groups, since he was required to assess drug compliance as per the trial protocol.

### Anesthesia

None of the patients received any preoperative medication. Standard monitoring (blood pressure, electrocardiogram, and pulse oximetry) was performed. In addition, to ensure maintenance of adequate anesthetic depth, the bispectral index (BIS™, Covidien Inc., USA) was recorded. Anesthesia was induced with intravenous pentothal (5 mg/kg), sevoflurane (Sevofran®, HANA PHARM Co., Ltd., South Korea) or desflurane (Suprane®, Baxter Health Corporation, USA), remifentanil (Ultiva®, GlaxoSmithKlein, UK) with a target-controlled infusion device (Orchestra®, Fresenius Vial, France), and rocuronium (0.6 mg/kg). For ventilation support, a laryngeal mask airway (i-gel®, Intersurgical Ltd., UK) or endotracheal tube was placed. Furthermore, mechanical ventilation was controlled to maintain end-tidal carbon dioxide at 33–38 mmHg. A continuous infusion of remifentanil and a volatile agent (sevoflurane or desflurane) was used to maintain anesthesia. Ringer’s lactate solution was infused at a rate of 5 mL/kg/h.

For postoperative pain control, either fentanyl 50 μg (when the verbal numerical rating scale (VNRS) > 5; VNRS 0 = “no pain” and VNRS 100 = “worst pain imaginable”) or ketorolac 30 mg (when the VNRS ≤ 5) was administered intravenously.

### PONV control (in-hospital)

After surgery, patients who suffered from nausea or vomiting were administered ramosetron 0.3 mg intravenously at the first episode. For subsequent episodes of nausea or vomiting, a single dose of 10 mg metoclopramide was given intravenously. Nausea was defined as a subjective feeling of being sick, and vomiting was defined as an expulsion of gastric contents or retching. This protocol was applied in both the patient groups.

### PDNV control (after discharge)

#### Ramosetron group

In the ramosetron group, ramosetron ODT 0.1 mg was administered on the morning of postoperative days 1 and day 2 as a prophylactic antiemetic after discharge. If there was nausea and/or vomiting before or after the administration of ramosetron ODT, metoclopramide 10 mg was administered as a rescue antiemetic, at a maximum dose of 30 mg per day.

#### Control group

In the control group, patients were administered only metoclopramide 10 mg for the treatment of nausea and/or vomiting at a maximum dose of 30 mg per day.

### Outcome measurements

The primary outcome was the incidence of nausea during the 24 h after discharge. The secondary outcomes included the incidence of nausea and vomiting (30 min and 3 h postoperatively, and 48 h after discharge), severity of nausea, and the rate of rescue antiemetic (metoclopramide) administration. Additionally, we investigated the pain scores and the dosage of analgesic used. The severity of nausea (0 = “no nausea” and 100 = “worst nausea imaginable”) and pain (0 = “no pain” and 100 = “worst pain imaginable”) was measured using the VNRS. The investigators interviewed the patients 24 and 48 h post-anesthesia by telephone.

### Sample size

By assuming that the incidence of post-discharge nausea was 30% (the overall incidence in patients who have two risk factors for PDNV) in the control group and 10% in the ramosetron group (about 70% reduction in the incidence of nausea from the control group), and that this difference was clinically significant, we calculated that 138 patients were needed to be recruited to achieve 80% power to detect this difference at a two-sided alpha level of 0.05, considering a 10% drop out rate.

### Statistical analysis

After normality check of variables using the Shapiro-Wilk test, continuous variables were compared with Student’s *t* test or Mann-Whitney *U* test and expressed as the mean (standard deviation (SD)) or median (interquartile range (IQR)), as appropriate. For categorical data, a chi-square test or Fisher’s exact test was used, and the results were described as a number (proportion). All analyses were carried out using IBM® SPSS® Statistics version 25.0 (IBM Corporation, NY, USA). *P* < 0.05 was considered as statistically significant.

## Results

**Fig. 1:**
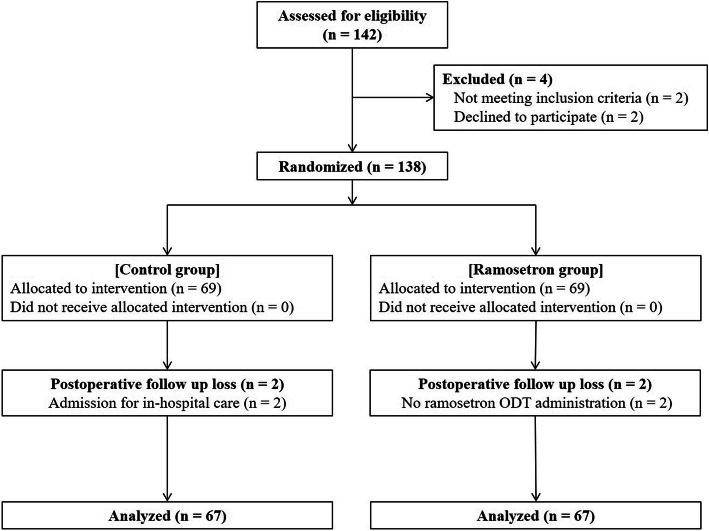
Flowchart for patient selection

A total of 142 patients were evaluated for eligibility of which 134 patients were finally analyzed. The reasons for exclusion from the analysis after group allocation were as follows: (1) two patients did not take ramosetron ODT according to the study protocol and (2) two patients were admitted for in-hospital care (Fig. 1). The characteristics of patients, surgery, and anesthesia are shown in Table 1.

**Table 1:** The characteristics of patients, surgery, and anesthesia

	Control (*n* = 67)	Ramosetron (*n* = 67)	*P*
Age (years)	39 (8)	40 (8)	0.344
Weight (kg)	61 (14)	58 (11)	0.257
Height (cm)	160 (5)	160 (5)	0.995
BMI (kg/m^2^)	23 (5)	22 (4)	0.175
ASA			0.136
1	42 (63%)	50 (75%)	
2	25 (37%)	17 (25%)	
Anesthesia time (min)	52 (23)	48 (21)	0.219
Surgery time (min)	27(19)	23 (17)	0.198
Previous PONV	1 (1%)	3 (4%)	0.619
Smoking	2 (3%)	4 (6%)	0.680
Types of surgery			0.587
Gynecology	52 (78%)	45 (67%)	
ENT	7 (10%)	9 (14%)	
Breast	3 (5%)	5 (7%)	
Orthopedics	2 (3%)	5 (7%)	
Dental	2 (3%)	3 (5%)	
Urology	1 (1%)	0 (0%)	

Data are expressed as mean (SD) or number of the patients (proportion)

*BMI* body mass index, *ASA* American Society of Anesthesiologists, *PONV* postoperative nausea and vomiting, *ENT* ear, nose, and throat

### Nausea and vomiting

The data regarding the PDNV are described in Table 2. We found that there were significant differences in the incidence (13% vs. 33%, *P* = 0.008) and severity (VNRS 5 vs. VNRS 14, *P* = 0.011) of nausea 24 h post-discharge between the ramosetron and control groups. In addition, the rate of rescue antiemetic (metoclopramide) administration during the 24 h after discharge was lower in the ramosetron group (6%) than in the control group (18%) (*P* = 0.033). During 24–48 h post-discharge, the incidence of nausea was not significantly different between the control (13%) and ramosetron (8%) groups (*P* = 0.259). The incidence of vomiting during the 24–48 h after discharge was comparable between the two groups (*P* = 1.000).

**Table 2:** Postoperative nausea and vomiting in hospital and after discharge

	Control (*n* = 67)	Ramosetron (*n* = 67)	*P* value
Postoperative 0–0.5 h			
Nausea	9 (13%)	4 (6%)	0.144
Vomiting	2 (3%)	0 (0%)	0.496
Nausea, VNRS	8 (22)	3 (11)	0.110
IV ramosetron	5 (8%)	1 (2%)	0.208
Postoperative 0.5–3 h			
Nausea	16 (24%)	17 (25%)	0.841
Vomiting	1 (2%)	2 (3%)	1.000
Nausea, VNRS	7 (18)	6 (13)	0.736
IV ramosetron	3 (5%)	2 (3%)	1.000
IV metoclopramide	2 (3%)	0 (0%)	0.496
Post-discharge 24 h			
Nausea	22 (33%)	9 (13%)	0.008
Vomiting	1 (2%)	2 (3%)	1.000
Nausea, VNRS	14 (23)	5 (16)	0.011
Oral metoclopramide	12 (18%)	4 (6%)	0.033
Oral metoclopramide (mg)	2.4 (5.8)	0.6 (2.4)	0.021
Post-discharge 24–48 h			
Nausea	9 (13%)	5 (8%)	0.259
Vomiting	0 (0%)	1 (2%)	1.000
Nausea, VNRS	5 (14)	4 (15)	0.673
Oral metoclopramide	4 (6%)	1 (2%)	0.365
Oral metoclopramide (mg)	0.9 (4.2)	0.1 (1.2)	0.162
Patient satisfaction score	86 (16)	95 (9)	<0.001

Data are expressed as mean (SD) or number of the patients (proportion)

*VNRS* Verbal numerical rating scale

### Other outcomes

Postoperative pain scores during the first 3 h after surgery were not significantly different between the two groups (Table 3). In addition, the requirement of analgesics was not significantly different between the two groups (Table 3). Patient satisfaction score regarding the management of PDNV was higher in the ramosetron group than in the control group (Table 2, *P* < 0.001).

**Table 3:** Postoperative pain and the amount of rescue analgesics

	Control (*n* = 67)	Ramosetron (*n* = 67)	*P* value
Postoperative 0–0.5 h			
VNRS	26 (21)	23 (18)	0.565
Fentanyl (μg)	9 (19)	6 (15)	0.335
Postoperative 0.5–3 h			
VNRS	15 (12)	14 (11)	0.497
Ketorolac (mg)	11 (14)	10 (14)	0.789

Data are expressed as mean (SD)

*VNRS* Verbal numerical rating scale

## Discussion

This multicenter randomized study showed that ramosetron ODT could reduce the incidence and severity of nausea with higher patient satisfaction, during the first 24 h after discharge. To the best of our knowledge, this is the first study on the prophylactic effect of ramosetron ODT on PDNV.

PDNV is a common complication but is rarely self-reported by patients after discharge, which explains why doctors and nurses often do not take it seriously. Carroll et al. (Carroll et al. 1995) found that the majority of patients did not have PONV during their stay in the hospital but experienced nausea a few hours later (usually within the first 24 h) at home. However, patients were able to manage PDNV with little help from health professionals and minimal use of medication. This is the reason why ramosetron ODTs were administered after discharge for home use, and not preoperatively. In addition, in the present study, the number of patients who needed the rescue antiemetic (metoclopramide) was greater in the control group (12 patients) than in the ramosetron group (4 patients) during the first 24 h after discharge, which supports the usefulness of ramosetron ODT at home to reduce PDNV.

In a study of outpatients, a history of PONV, age < 50 years, female sex, nausea in the post-anesthesia care unit (PACU), and opioid use in the PACU were identified as independent predictors of PDNV, and the overall incidence of PDNV was found to be 37% in the first 48 h after discharge (Apfel et al. 2012). The incidence of PDNV in the presence of none to all five of these risk factors was estimated to be about 10%, 20%, 30%, 50%, 60%, and 80%, respectively (Apfel et al. 2012).

In 2011, Melton et al. (2011) reviewed the management of PDNV after outpatient surgery and concluded that an effective PDNV regimen included traditional intraoperative PONV prophylaxis in combination with post-discharge oral 5-HT_3_ receptor antagonists. However, few clinical studies have assessed the effect of oral antiemetics on PDNV since their review. Our data support their recommendation that an oral 5-HT_3_ receptor antagonist may be considered as one of the agents for combination antiemetic therapy for PDNV.

In the present study, ramosetron ODT lowered the incidence of nausea from 33 to 13% for 24 h after discharge. So far, most studies investigating the prophylactic effect of oral 5-HT_3_ receptor antagonists on PDNV have used ondansetron ODT. The first study on the effect of ondansetron ODT on PDNV was performed by Gan et al. (2002); they included patients undergoing outpatient gynecological laparoscopy under general anesthesia. Patients who took ondansetron ODT 8 mg experienced less severe nausea and fewer vomiting episodes after discharge than patients on placebo. Pan et al. (2008) conducted a study on high emetic-risk outpatients to assess the efficacy of two types of antiemetic prophylaxes in preventing PDNV and the impact on their quality of life during the recovery period, i.e., between days 1 and 5 after discharge. In patients who were administered additional intraoperative dexamethasone and oral ondansetron once a day, a significant reduction in the incidence of PDNV and improvement in quality of life were observed compared with a single dose of intraoperative IV ondansetron prophylaxis. Another clinical study was performed by Kim et al. (Kim et al. 2013) to assess the prophylactic effect of ondansetron ODT, administered at home, on PDNV in patients who underwent hip arthroplasty under neuraxial anesthesia. In contrast to the study conducted by Pan et al. (2008), this study did not confirm the efficacy of prophylactic ondansetron administration at home. On the first day post-discharge, 54% of the placebo group and 46% of the oral ondansetron group experienced nausea. On days 2 and 3 post-discharge, 16% and 11% of the placebo group experienced nausea, while 18% and 10% of the oral ondansetron group experienced nausea, respectively.

Although there have been clinical trials investigating the effect of ramosetron ODT on PONV, there is no data on PDNV. Ryu et al. (2011) conducted the following double-blinded study in 2011. Patients scheduled for laparoscopic cholecystectomy were allocated to 1 of 3 groups: 0.3 mg intravenous ramosetron, 0.1 mg oral ramosetron, or a combination of oral and intravenous ramosetron. For the prophylaxis of nausea and vomiting after laparoscopic cholecystectomy during the first 24 h after surgery, the combination of intravenous and oral ramosetron was more effective than either intravenous ramosetron or oral ramosetron alone. In another study, preoperative oral administration of ramosetron at a dose of 0.1 mg was found to be an acceptable and effective method of reducing the incidence of PONV in breast cancer patients (Lee et al. 2008). When compared to the no prophylaxis group (75.3%), the overall incidence of nausea and vomiting during the first 24 h after recovery was significantly lower in the intravenous ramosetron (27.8%) and ramosetron ODT (25%) groups.

Our study has some limitations. First, the investigator performing the outcome assessment and the patients were not blinded. Thus, there could have been unidentified confounders; however, the assessment was conducted using a uniform interview sheet, which might have reduced the chance of bias during the interview. In addition, most patients took the study drug as per protocol, and those who did not were excluded from the subject. Second, since it was performed in high-risk patients (female, aged < 50 years old, and operated under general anesthesia), the generalizability of the results may be limited.

In conclusion, ramosetron ODT reduces the incidence and severity of nausea during the first 24 h after discharge and may be a valuable option for the prevention of PDNV in female patients after day surgery under general anesthesia.

## Data Availability

The data that support the findings of this study are available from the corresponding author upon reasonable request.

## Abbreviations

PACU: Post-anesthesia care unit
PONV: Postoperative nausea and/or vomiting
PDNV: Post-discharge nausea and/or vomiting
ODT: Orally disintegrating tablet
5-HT: 5-Hydroxytryptamine
VNRS: Verbal numerical rating scale
